# Role of ferroptosis and its non-coding RNA regulation in hepatocellular carcinoma

**DOI:** 10.3389/fphar.2023.1177405

**Published:** 2023-04-13

**Authors:** Lei Yang, Yu Guan, Zhanbing Liu

**Affiliations:** Department of General Surgery, Peking University First Hospital, Beijing, China

**Keywords:** hepatocellular carcinoma, ferroptosis, non-coding RNA, treatment, prognosis

## Abstract

Ferroptosis is a newly discovered form of programmed cell death that involves the accumulation of iron-dependent lipid peroxides and plays a vital role in the tumorigenesis, development, and drug resistance of various tumors such as hepatocellular carcinoma (HCC). As a hotspot in molecular biology, non-coding RNAs (ncRNAs) participate in the initiation and progression of HCC, either act as oncogenes or tumor suppressors. Recent studies have shown that ncRNAs can regulate ferroptosis in HCC cells, which would affect the tumor progression and drug resistance. Therefore, clarifying the underlying role of ferroptosis and the regulatory role of ncRNA on ferroptosis in HCC could develop new treatment interventions for this disease. This review briefly summarizes the role of ferroptosis and ferroptosis-related ncRNAs in HCC tumorigenesis, progression, treatment, drug resistance and prognosis, for the development of potential therapeutic strategies and prognostic markers in HCC patients.

## Introduction

**FIGURE 1:**
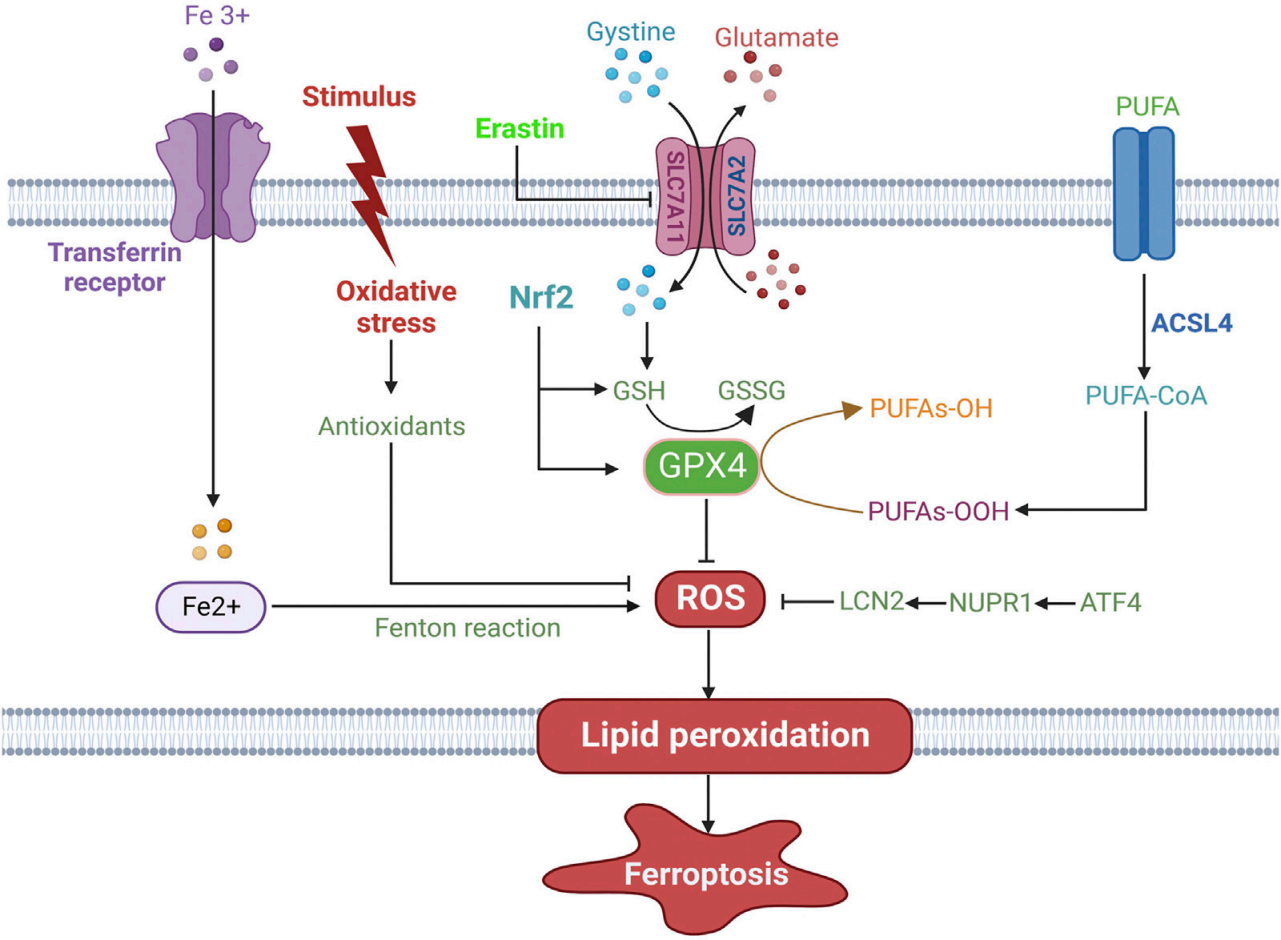
The mechanisms of ferroptosis in HCC. Ferroptosis is executed by generation of ROS from iron accumulation through the Fenton reaction and oxidative stress, which induces lipid peroxidation and ferroptotic cell death. Several antioxidant systems, including the SLC7A11/GSH/GPX4 system, the ATF4/NUPR1/LCN2 signaling pathway, Nrf2 and oxidative stress-activated antioxidants, inhibit ferroptosis. Among them, GPX4 is a crucial molecule and converts the toxic PUFA-OOH to non-toxic PUFA-OH, thus suppressing the occurrence of ferroptosis. PUFA is catalyzed by ACSL4 and is further oxidized to PUFA-OOH, which contributes to lipid peroxidation the cell membrane. ROS: reactive oxygen species; SLC7A11: solute carrier family 7 member 11; GSH: glutathione; GPX4: glutathione peroxidase 4; ATF4: activating transcription factor 4; LCN2: lipocalin 2; Nrf2: nuclear factor E2 related factor 2; PUFA: polyunsaturated fatty acid; ACSL4: acyl-CoA synthetase long-chain family member 4. → indicates a promoting effect and ⊥ indicates an inhibitory effect.

Hepatocellular carcinoma (HCC) is the second leading cause of cancer-related death worldwide in men, with high morbidity and high-grade malignancy (Llovet et al., 2021). Surgery is a curative intervention for patients with early HCC and sorafenib, a multikinase inhibitor, is the first-line chemotherapeutic drug for advanced HCC, but chemoresistance in these patients results in tumor recurrence and metastasis (Singh et al., 2022). Ferroptosis, a relatively new form of programmed cell death caused by overwhelming lipid peroxides that derived from iron metabolism, is considered to induce fatal cell damage and becomes an emerging cancer suppression mechanism for HCC (Si et al., 2022). The process of ferroptosis mainly includes iron metabolism, reactive oxygen species (ROS) accumulation, lipid peroxidation, and redox disruption, all of which is intricately regulated by a variety of signals, such as the SLC7A11 (solute carrier family 7 member 11)/GSH (glutathione)/GPX4 (glutathione peroxidase 4) and NRF2 (nuclear factor E2 related factor 2) signaling pathways (Figure 1), which is discussed in recent comprehensive reviews (Capelletti et al., 2020; Xu C. et al., 2021). This process is focused on HCC research owing to its involvement in tumorigenesis, progression, diagnosis, chemoresistance, and prognosis (Wang K. et al., 2021; Liu et al., 2022). Understanding the role of ferroptosis in HCC is of great significance, and regulation of ferroptosis represents a new potential prognostic biomarkers and therapeutic target in this disease. It should be noted that the non-coding RNA (ncRNAs), mainly contain microRNA (miRNA), long non-coding RNA (lncRNA), and circular RNA (circRNA), play a crucial role in the occurrence and development of HCC (Wong et al., 2018). Mechanically, miRNAs directly target mRNAs to influence the gene translation, and both lncRNAs and circRNAs can sponge miRNAs to regulate the miRNAs-mediated gene regulation; moreover, ncRNAs directly bind to proteins and thus block proteins’ functions and downstream signaling pathways (Liu et al., 2021). A growing body of evidence indicate that ncRNA-regulated ferroptosis is associated with malignant behaviors like proliferation, metastasis and chemoresistance in HCC (Qi et al., 2019; Bai et al., 2020; Xu et al., 2020). Identification of the regulatory roles of ncRNAs on ferroptosis in the progression and treatment of HCC could provide promising therapeutic strategies and prognostic markers for this disease. This review summarizes the role of ferroptosis in HCC development, treatment, chemoresistance and prognosis in this disease. It also highlights the latest research progression on the role of ncRNAs-regulated ferroptosis in HCC, and discusses the potential of ferroptosis-regulating ncRNAs as therapeutic targets.

## Ferroptosis in HCC

### Proliferation, migration and invasion

The proliferation, invasion, and metastasis of cancer cells are vital events in the occurrence and development of malignancies. Increasing studies investigating the relationship of ferroptosis and HCC have unveiled that ferroptosis serves as a regulator of HCC through affecting the biological characteristics of cancer cells, such as proliferation, migration, and apoptosis (Table 1). For instance, protocadherin beta 14 (CDHB14), is downregulated in HCC patients, represses cell proliferation by promoting ferroptosis via the blockage of p65/SLC7A11interaction, suggesting a tumor-suppressing role of CDHB14 in HCC (Liu et al., 2022). However, several overexpressed proteins in HCC are associated with ferroptosis and tumorigenesis. Zhang et al. demonstrated that the zinc-finger protein 498 (ZNF498), which indicates advanced pathological grade and unfavorable prognosis in patients with HCC, accelerates hepatocarcinogenesis and progression, as well as impedes the p53-triggered apoptosis and ferroptosis (Zhang B. et al., 2022). Glucose-6-phosphate dehydrogenase (G6PD) is verified to be an independent risk factor related to the adverse outcomes of HCC, and exacerbates tumor growth, invasion, and metastasis, along with reduced ferroptosis through suppressing cytochrome P450 oxidoreductase (POR) (Cao et al., 2021). These findings imply that inducing ferroptosis may be employed as a therapeutic strategy for HCC. Indeed, targeting ferroptosis suppressors has been implicated in the treatment of HCC. It is reported that silencing of centrosomal protein 290 (CEP290) restrains HCC growth and progression but facilitates ferroptosis through activating the NRF2 signaling pathway (Shan et al., 2022). Likewise, ENO1 (alpha-enolase), as an RNA-binding protein, abrogates the expression of iron regulatory protein 1 (IRP1) and mitochondrial iron-induced ferroptosis, indicating an underlying therapeutic target for HCC (Zhang T. et al., 2022). Under hypoxic conditions, HCC can develop resistant mechanisms in response to anticancer therapies. The depression of methyltransferase-like 14 (METTL14) induced by hypoxia blocks ferroptosis and thus reduces the efficacy of HCC interventional embolization, resulting from SLC7A11 degradation induced by YTH N6-methyladenosine RNA binding protein (YTHDF) (Fan et al., 2021). Another deep impressed investigation demonstrated that knockdown of isocitrate dehydrogenase 2 (IDH2), a nicotinamide adenine dinucleotide phosphate (NADPH)-producing enzyme, improves erastin-induced ferroptosis in HCC cells by reducing mitochondrial GSH, and then inhibits HCC progression (Kim et al., 2020). The above studies show that ferroptosis plays a crucial role in the initiation and progression of HCC, and its specific role and mechanism need to be further explored. Besides, ferroptosis also participates in angiogenesis of HCC. It is reported that the miR-17-92 cluster in cultured human HCC cells enhances cell proliferation, colony formation, and invasiveness (Zhu et al., 2015). As an oncogenic miRNA cluster, miRNA-17-92 protects endothelial cells from erastin-induced ferroptosis by downregulating the expression of ACSL4 (Xiao et al., 2019), thus promoting tumor angiogenesis in HCC. Hence, targeting ferroptosis regulators may provide novel strategies for HCC treatment.

**TABLE 1:** Candidate substances and genes for inducing ferroptosis in HCC.

Substances and genes	Target	Mechanism	Experimental model	Ref.
CEP290	Nrf2	CEP290 depletion inhibit HCC progression by elevating Fe^2+^ and malondialdehyde levels	Hep3B cell lines, human HCC tissues	Shan et al. (2022)
PCDHB14	SLC7A11	Inhibits HCC proliferation and induces ferroptosis	Diethylenenitrite-induced HCC mice model, human HCC tissues	Liu et al. (2022)
ZNF498	p53	Suppresses HCC apoptosis and ferroptosis by attenuating p53 phosphorylation	Diethylenenitrite-induced HCC mice model, human HCC tissues	Zhang B. et al. (2022)
ENO1	IRP1	Promote HCC survival by repressing mitochondrial iron-induced ferroptosis	Huh7 and HepG2 cell lines, human HCC tissues, nude mice	Zhang T. et al. (2022)
METTL14	SLC7A11	METTL14 inhibition by hypoxia facilitate HCC progression via abrogating ferroptosis	Huh7, HepG2, 7721, HCCLM3, MHCC97H, PLC/PRF/5 and Bel-7402 cell lines, nude mice	Fan et al. (2021)
G6PD	POR	Promotes HCC progression via inhibiting ferroptosis	HepG2, Hep3B217 and SNU387 cell lines; nude mice	Cao et al. (2021)
IDH2	Unknown	IDH2 knockdown increases susceptibility to erastin-induced ferroptosis in HCC	Hepa1-6 cell lines, nude mice	Kim et al. (2020)
IFN γ	SLC3A2, SLC7A11	Sensitizes HCC to ferroptosis and increases ROS production	Bel7402 and HepG2 cell lines	Kong et al. (2021)
Compound 21	GPX4, ACSL4	Blocks the cell cycle and induces HCC ferroptosis	HepG2 and H22 cell lines, human HCC tissues, nude mice	Wang H. et al. (2022)
Dihydroartemisinin	PEBP1, CHAC1, SLC7A11	Induces HCC ferroptosis and inhibits tumor growth, and enhances the tumor-suppressing effect of sorafenib	Huh-7 and HepG2 cell lines, nude mice	Su et al. (2021)
				Wang et al. (2021b)
				Cui et al. (2022)
Rhamnazin	GPX4	Inhibits HCC progression through inducing ferroptosis and ROS accumulation	Huh7 and SMMC-7721 cell lines	Mei et al. (2022)
Heteronemin	GPX4	Induces HCC death by inducing ferroptosis and ROS formation	HA22T and HA59T cell lines	Chang et al. (2021)
Solasonine	GPX4, GSS	Inhibits HCC progression via promoting ferroptosis and ROS production	HepG2 and HepRG cell lines	Jin et al. (2020)

In sum, the dysregulation of iron metabolism and ferroptosis is involved in HCC progression. Emerging ferroptosis-related regulators have been demonstrated to modulate ferroptosis in HCC, such as polypyrimidine tract binding protein and protocadherin (Jun et al., 2023; Yang et al., 2023). However, the regulatory mechanism of ferroptosis-related mediators and its downstream signaling are still vague. Besides, as programmed cell death processes, both apoptosis and ferroptosis are crucial cell death mechanisms that are effective on cancer treatment. Ferroptosis is an innovative opportunity for treatment in the era of apoptosis resistance. Indeed, the NRF2 is verified to act as a hub pathway to mediate ferroptosis and apoptosis in HCC, suggesting the synergistic interaction between ferroptosis and apoptosis (Li et al., 2023). Currently, the relationship between ferroptosis and apoptosis is unclear in HCC. The unfolded protein response and subsequently endoplasmic reticulum stress-activated signaling pathways participate in the crosstalk between ferroptosis and apoptosis during cancer progression (Lee et al., 2018). Elaborating the intrinsic signaling cascades is vital for understanding the pathogenesis and developing novel treatments of HCC. Once apoptosis resistance is formed in HCC, targeting these cellular signaling to switch apoptosis to ferroptosis has a great therapeutic potential in this disease.

### Tumor microenvironment and immunotherapy

The tumor microenvironment (TME) is a well-organized ecosystem, that is, composed of malignant cells, immune cells, fibroblasts, and vascular endothelial cells. As the main components to interact with malignant cells, immune cells can affect tumorigenesis, progression, metastasis, and treatment resistance (Tang B. et al., 2023). It is generally acknowledged that HCC is characterized by a highly suppressive tumor immune microenvironment, which is responsible for the immune escape of cancer cells from the detection and elimination by host immunosurveillance (Llovet et al., 2022). Thus, regulation of TME is a crucial target for HCC treatment. Ferroptosis seems to play a dual role in the TME of HCC. It not only restrains the activity of antitumor immune cells and compromises the antitumor immunity, but causes the reversal of immunosuppression through the functional regulation of immunosuppressive immune cells (Cong et al., 2022). It is reported that ferroptosis-related genes are associated with infiltration of protumor immune cells and expressions of inhibitory checkpoint molecules, indicating the involvement of ferroptosis in construction of the immunosuppressive TME in HCC (Hu Y. et al., 2022). Similarly, HBV infection facilitates HCC cells to secrete the exosome miR-142-3p, which decreases the expression of SLC3A2 and further induces ferroptosis in M1 macrophages, accelerating the progression of HCC (Hu Z. et al., 2022). Hence, protection of antitumor immune cells from ferroptosis may halt HCC development. Besides, suppression of apolipoprotein C1, a key protein in lipid metabolism, can induce M1 polarization via ferroptosis pathway, which reshapes the immunosuppressive TME and enhances anti-PD1 immunotherapy for HCC (Hao et al., 2022). Thereby, inhibiting ferroptosis in M1 macrophages may exert antitumor immunity in HCC. Furthermore, as an immunomodulatory cytokine with antiviral and antitumor functions, interferon-γ (IFN-γ) promotes erastin-induced ferroptosis by mediating mitochondrial dysfunction and improving ROS leakage in HCC (Kong et al., 2021). Therefore, modulation of ferroptosis can ameliorate the tumor immunosuppressive microenvironment and sensitize HCC to immunotherapy.

Immunotherapy has a promising therapeutic effect on malignant tumors, especially, immune checkpoint inhibitors (ICIs) have shown potent antitumor efficacy in advanced melanoma or lung cancer via repressing inhibitory pathways including PD-1/PD-L1 and CTLA-4 in effector T cells. Although ICIs present satisfactory toxicity and safety in patients with advanced HCC, they only have a response rate of less than 20% (El-Khoueiry et al., 2017; Zhu et al., 2018), owing to the immunosuppressive microenvironment. Thus, targeting ferroptosis to orchestrates the TME will provide novel immunotherapeutic strategies for HCC. Indeed, several prediction models are established based on differential expression levels of ferroptosis-related genes to estimate TME status and immunotherapy efficacy in patients with HCC (Xu et al., 2022; Yang C. B. et al., 2022). According to sample analyses, patients are categorized into high-risk and low-risk groups, with high-risk groups exhibiting shorter overall survival and unfavorable prognosis; moreover, the high-risk groups show more immunosuppressive cells, such as macrophages, Th2 cells, and Tregs infiltration, as well as increased expression of immune checkpoints, implying that patients in high-risk groups may show satisfactory response to ICIs therapies. In addition, the ferroptosis inducer erastin is verified to affect Th17 cell differentiation and IL-17 signaling pathway, which has a therapeutic potential in HCC immunotherapy (Tang et al., 2020). A recent study has unveiled that knockout of SLC7A11, a ferroptosis-related gene, in macrophage reduces the recruitment and infiltration of tumor-associated macrophages by activating ferroptosis, which elevates PD-L1 expression in macrophages and improves the antitumor efficacy of anti-PD-L1 therapy (Tang B. F. et al., 2023). Also, several novel therapies based on inducing ferroptosis in HCC cells have been demonstrated to enhance antitumor immunity, along with increased activated CD8^+^ T cells and matured dendritic cells but decreased myeloid-derived suppressor cells in tumor tissues (Xu Q. et al., 2021; Chen et al., 2022a).

In short, modulating ferroptosis to orchestrates the TME is a promising strategy for improvement in immunotherapeutic efficacy of HCC. However, due to the unsatisfactory response rates of immunotherapy in patients with advanced HCC, novel markers should be clarified to assess who may benefit from ferroptosis combined with immunotherapy. Besides, considering the intricate crosstalk between the ferroptosis and TME, the association between ferroptosis and immunotherapy should also be determined according to various immunophenotypes, regulatory mechanisms of ferroptosis, and types of immune cells.

### Antitumor intervention

Ferroptosis is regarded as a potential mechanism mediating antitumor activity. Emerging studies endeavor to identify promising candidates for HCC treatment that involved in ferroptosis induction (Table 1). Compound 21, a derivative of seco-lupane triterpene, triggers ferroptosis and blocks cell cycle, thus mediating the death process of HCC cells, which suggests that the induction of ferroptosis may be the main mechanism eliciting the antitumor effect (Wang H. et al., 2022). Another substance, Rhamnazin, can inhibit tumor proliferation and invasion by suppressing GPX4 expression and further upregulating the level of lipid peroxides, iron and ROS (Mei et al., 2022). The data of Chang et al. implied that heteronemin, a marine terpenoid, could induce HCC ferroptosis and apoptosis through decreasing the GPX4 and activating the MAPK pathway (Chang et al., 2021). The results show that Solasonine performs as a potential novel compound for HCC treatment via promoting ferroptosis via GPX4-mediated disruption of the glutathione redox system (Jin et al., 2020). Dihydroartemisinin (DHA), a pharmacologically active component isolated from artemisinin, induces ferroptosis in HCC cells by elevating the expression of phosphatidylethanolamine Binding Protein 1 (PEBP1) and the level of lipid peroxidation (Su et al., 2021). Further mechanistic investigations explained that DHA-induced ferroptosis is associated with unfolded protein response-mediated upregulation of chaC glutathione specific gamma-glutamylcyclotransferase 1 (CHAC1), a GSH-degrading gene (Wang et al., 2021b). Therefore, combination DHA and sorafenib has a synergistic inhibitory effect on HCC cells by attenuated energy metabolism and potent ferroptosis, which is evident by increased levels of lipid ROS, iron and MDA, as well as reduced expression of GSH, SLC7A11 and GPX4 (Cui et al., 2022). To investigate the efficacy of IFN-γ on HCC treatment, Kong et al. concluded that IFN-γ administration could sensitize HCC cells to ferroptosis through repressing system xc^−^ activation via stimulating the JAK/STAT signaling pathway (Kong et al., 2021). Thus, ferroptosis activators provide new insights for HCC treatment.

Thus, some drugs and natural compounds have been implicated in the activation of ferroptosis and play a tumor-suppressive role in the progression of HCC. Further clarification of the mechanisms by which various drugs or natural products regulate ferroptosis is crucial to develop targeted interventions in HCC. However, whether various targets acting on the ferroptosis exist in these drugs or natural products needs to be confirmed. Otherwise, the effect of most of the ferroptosis inducers has been studied in both cell experiments and animal models with no measurable side effects. The efficacy of these drugs should be further evaluated in clinical settings for the treatment of HCC, and a more comprehensive evaluation of the side effects of these drugs should be performed in the future.

### Drug resistance

**FIGURE 2:**
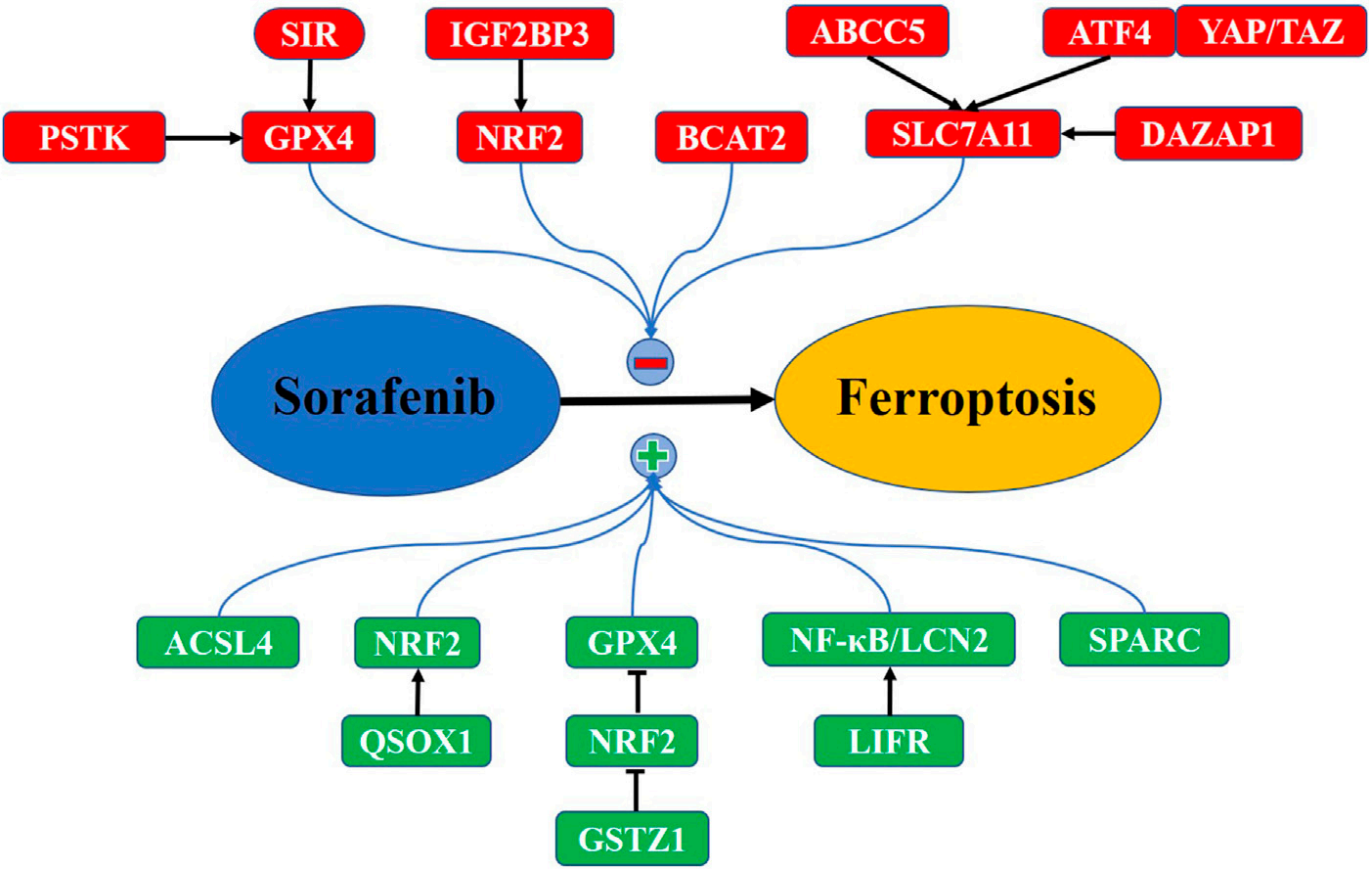
Key pathways in modulation of sorafenib-mediated ferroptosis in HCC. Some negative regulators (red) inhibit sorafenib-mediated ferroptosis, which induces HCC resistance to sorafenib. Other positive regulators (green) promote sorafenib-mediated ferroptosis, sensitizing HCC to sorafenib (see text).

The drug resistance of patients with HCC largely affects the efficacy of chemotherapy. As a ferroptosis inducer, sorafenib is an essential chemotherapeutic drug for HCC. Recent studies have suggested that many ferroptosis regulators could affect the sensitivity of HCC cells to sorafenib (Figure 2). Feng et al. (2021) demonstrated that acyl-CoA synthetase long-chain family member 4 (ACSL4), a ferroptosis-activating enzyme, enhances sorafenib-induced ferroptosis and efficiently predicts sorafenib sensitivity in HCC. The findings revealed that quiescin sulfhydryl oxidase 1 (QSOX1) renders HCC cells sensitive to oxidative stress and potentiates sorafenib-induced ferroptosis by suppressing NRF2, indicating an underlying therapeutic target for HCC (Sun et al., 2021). Silencing of insulin-like growth factor 2 mRNA-binding protein 3 (IGF2BP3), an N6-methyladenosine reader, results in the instability of NRF2 mRNA, and thus overcomes resistance to sorafenib via promoting ferroptosis in HCC (Lu Y. et al., 2022). The NRF2/GPX4 axis is also inhibited by glutathione S-transferase zeta 1 (GSTZ1, a phenylalanine-metabolizing enzyme) in sorafenib-resistant HCC cells, subsequently lipid peroxidation and ferroptosis is promoted, which sensitizes HCC cells to sorafenib (Wang et al., 2021c). Moreover, suppression of PSTK (phosphoseryl-tRNA kinase), promotes ferroptosis and improves the effect of sorafenib in HCC through inactivating the GPX4 and further disrupting the glutathione metabolism (Chen et al., 2022b). Besides, upregulation of the secreted protein acidic and rich in cysteine (SPARC) the cytotoxicity of sorafenib in HCC by elevating the level of ROS and further activating ferroptosis (Hua et al., 2021). Therefore, these results indicate that ferroptosis activators combined with sorafenib exhibit synergistic efficacy in HCC treatment, which might be an enlightening therapeutic tactic for this disease.

However, several negative ferroptosis regulators are demonstrated to induce the chemoresistance to sorafenib in HCC. For instance, ABCC5 (ATP binding cassette subfamily C member 5), highly expressed in sorafenib-resistant HCC cells, elevates GSH and alleviates lipid peroxidation by stabilizing SLC7A11, thus leading to suppression of ferroptosis and inducing the resistance to sorafenib (Huang et al., 2021). Similarly, DAZAP1 (deleted in azoospermia associated protein 1), correlated with poor clinical prognoses in HCC, can inhibit ferroptosis via binding to SLC7A11 mRNA, which decreases the sensitivity of HCC cells to sorafenib (Wang Z. et al., 2021). Gao L. et al. (2021) illuminated that YAP/TAZ cooperates with ATF4 (activating transcription factor 4) to activate the expression of SLC7A11, thereby enabling HCC cells to overcome sorafenib-mediated ferroptosis. These results imply that SLC7A11 can be targeted by ferroptosis-related regulators to reduce sorafenib resistance in HCC. Furthermore, the branched-chain amino acid aminotransferase 2 (BCAT2), is upregulated in HCC tissues and prevents cancer cells from sorafenib-induced ferroptosis by increasing glutamate; however, this effect is blocked by inhibiting system Xc-activity (Wang K. et al., 2021). It is reported that sigma-1 receptor (S1R) is overexpressed in sorafenib-treated HCC, and its depletion drives ferroptosis via inhibiting the expression of GPX4, along with increased iron metabolism, lipid peroxidation, and sorafenib sensitivity (Bai et al., 2019). Analogously, depression of LIFR (leukemia inhibitory factor receptor) initiates hepatocarcinogenesis and confers HCC cell resistance to sorafenib-induced ferroptosis by activating the NF-κB/LCN2 (lipocalin 2) activity (Yao et al., 2021). Thus, targeting ferroptosis-suppressors could rescue HCC cell resistance to sorafenib.

In conclusion, some ferroptosis-related regulators are expressed differently in HCC and affect the efficacy of sorafenib, either acting as a tumor-suppressor or oncogene by regulating the ferroptosis process, including oxidative stress, iron overload, and lipid peroxidation. Therefore, downregulating oncogenic regulators or rejuvenation of tumor-suppressive regulators in HCC patients with combination of sorafenib may provide a potential therapeutic strategy. Otherwise, as a crucial targeted therapeutic agent, lenvatinib also induces ferroptosis by suppressing the expression SLC7A11 and GPX4 in HCC; moreover, NRF2-overexpressing HCC cells display resistance to lenvatinib and low lipid ROS levels, suggesting lenvatinib-induced ferroptosis is regulated by NRF2 (Iseda et al., 2022). In this regard, further identification of intermediate factors to regulate ferroptosis-related downstream signaling pathways helps control levels of ferroptosis. Furthermore, some regulators linking ferroptosis to the tumor microenvironment that have implicated in HCC progression should be further investigated.

### Prognostic markers

Ferroptosis-related genes and proteins are expected to act as prognostic biomarkers in HCC. It is showed that PRDX1 (peroxiredoxin 1), a ferroptosis promoter, combines with GPX2, MT3 (membrane receptor 3) and SRXN1 (sulfiredoxin 1) to establish an effective prognosis model for HCC (Luo et al., 2022). In addition, TEAD2 (TEA domain transcription factor 2), a ferroptosis regulator, is demonstrated to function as novel prognostic target for the prediction of HCC outcome, and is associated with infiltrating immune cells including macrophages, neutrophils, and lymphocytes (Ren et al., 2022). Furthermore, increasing ferroptosis-related gene signature has been proved to predict HCC survival and treatment. For example, several ferroptosis-related prognostic models participate in the formation of immune microenvironment and tumor-related gene mutation, and are related to the overall and disease-specific survival in patients with HCC (Liang et al., 2020; Deng et al., 2021). Wan et al. (2022) screen 5 ferroptosis-related markers [G6PD, HMOX1 (heme oxygenase 1), LOX (lysyl oxidase), SLC7A11, and STMN1 (stathmin 1)], which are associated with higher tumor-node-metastasis stage, cirrhosis and immunosuppressive microenvironment in tumor tissue, and further well predict the prognosis of HCC. Studies have found that ferroptosis-related genes serve as independent risk factors to evaluate the clinical efficacy of chemotherapy and immunotherapy in HCC (Gao R. et al., 2021; Xiong et al., 2022). The establishment of markers associated with ferroptosis will be beneficial to predict the biological characteristics of HCC and select the optimal therapeutics for HCC patients. However, there are still many issues to be solved in the application of ferroptosis to the clinical diagnosis and treatment of HCC.

Altogether, the ferroptosis-related regulators are dysregulated in HCC and could be used as potential prognostic biomarkers to optimize patient monitoring and identify novel targets for more effective therapies. However, more attention should be given to precise research, such as changes and functions of the regulators based on different tumor stages and types in large samples.

## Ferroptosis-related ncRNAs in HCC

### Ferroptosis-regulating ncRNAs in HCC progression

Growing studies have unveiled that ferroptosis-related ncRNAs are involved in the initiation, progression, prognosis, and drug resistance of HCC. Some tumor-suppressive ncRNAs activate ferroptosis to impede HCC development and chemotherapeutic efficacy, whereas other oncogenic ncRNAs target ferroptosis-related components and facilitate tumorigenesis and progression of HCC (Table 2).

**TABLE 2:** Regulation of ncRNAs in ferroptosis of HCC.

NcRNA	Expression	Ferroptosis	Target	Outcome	Ref.
miR-214	Down	Promotion	ATF4	Increase the erastin-mediated tumor-suppressing effects	Bai et al. (2020)
miR-23a-3p	Up	Inhibition	ACSL4	Reduce sorafenib response	Lu Y. et al. (2022)
lncHEPFAL	Down	Promotion	SLC7A11	Increase the erastin-induced ferrotoptic cell death	Zhang X. et al. (2022)
lncHULC	Up	Inhibition	miR-3200-5p/ATF4	Faciliate HCC proliferation and metastasis	Guan et al. (2022)
lncNEAT1	Up	Promotion	miR-362-3p/MIOX	Increase the anti-tumor activity of erastin	Zhang Y. et al. (2022)
lncPVT1	Down	Promotion	miR-214-3p/GPX4	Involve in ketamine-mediated tumor suppression	He et al. (2021)
lncGABPB1-AS1	Up	Promotion	GABPB1	Inhibit the antioxidant capacity and viability of HCC	Qi et al. (2019)
circIL4R	Up	Inhibition	miR-541-3p/GPX4	Promote HCC progression	Xu et al. (2020)

ATF4 is regarded as a negative regulator in ferroptosis, and miR-214 is a potential inhibitor of ATF4. Bai et al. revealed that overexpression of miR-214 improves the levels of Fe^2+^, ROS and malondialdehyde by blocking ATF4, which renders HCC cells susceptible to erastin-mediated ferroptosis (Bai et al., 2020). It was found that the GABPB1, is highly expressed and related to unfavorable prognosis in HCC, and could be blocked by lncRNA GABPB1-AS1, which inhibits the expression of PRDX5 (peroxiredoxin 5) peroxidase and further attenuates the antioxidant capacity of HCC cells, thus leading to an enhancement of erastin-induced ferroptosis (Qi et al., 2019). The study of Zhang et al. confirmed that lncRNA HEPFAL plays a crucial role in promoting ferroptosis by decreasing the expression of SLC7A11, highlighting a therapeutic potential of lncRNA HEPFAL in HCC (Zhang X. et al., 2022). Intriguingly, lncRNA NEAT1 is upregulated when HCC cells are exposed to erastin, and functions as a decoy of miR-362-3p to enhance ROS production but reduce NADPH and GSH through elevating the expression of MIOX (myoinositol oxygenase), which intensifies ferroptosis in HCC cells (Zhang Y. et al., 2022). Ketamine can inhibit the proliferation and facilitates the apoptosis of HCC cells. Ketamine induces ferroptosis and represses malignant phenotypes of HCC cells via the lncRNA PVT1/miR-214-3p/GPX4 axis (He et al., 2021). These findings indicate that ncRNAs can act as promoters of ferroptosis, indicating a potential therapeutic strategy for HCC.

However, several studies have shown that ncRNAs play an oncogenic role in HCC by suppressing ferroptosis. Lu and co-workers conducted a study on chemotherapeutic implication of miR-23a-3p in sorafenib-resistant HCC cells. They showed that miR-23a-3p is overexpressed in sorafenib non-responders, suggesting unfavorable prognosis in HCC. Silencing of miR-23a-3p expedited sorafenib sensitivity of HCC via promoting ferroptosis. Further molecular investigation demonstrated that miR-23a-3p mediates sorafenib resistance through suppression of ACSL4 and thus reducing iron accumulation and lipid peroxidation (Lu Z. et al., 2022). Also, depletion of lncRNA HULC facilitates ferroptosis and oxidative stress in HCC cells via the miR-3200-5p/ATF4 Axis, leading to the repression of proliferation and metastasis in cancer cells (Guan et al., 2022). Likewise, Xu et al. investigated the effects of miR-541-3p on circIL4R-mediated effects on the progression of HCC cells. In their study, a remarkable circIL4R high expression is observed in HCC tissues in comparison with their corresponding normal samples. Depression of circIL4R abrogates oncogenesis and accelerates ferroptosis of HCC cells by sponging miR-541-3p to suppress the expression of GPX4, suggesting that circIL4R functions as a tumor promoter and a ferroptosis inhibitor in HCC via the miR-541-3p/GPX4 axis (Xu et al., 2020). These results imply that targeting ferroptosis-suppressive ncRNAs may provide a novel target in the treatment of HCC.

Collectively, aberrant expression of ncRNAs influences the initiation and development of HCC by regulating ferroptosis, thus identifying ferroptosis-related ncRNAs and the regulatory role of ncRNAs in ferroptosis will shed light on the pathogenesis and therapies of this disease. Several ncRNAs (miR-214, lncHEPFAL, lncNEAT1, lncPVT1, lncGABPB1-AS1) can induce ferroptosis to inhibit HCC progression, while other ncRNAs (miR-23a-3p, lncHULC, circIL4R) promote HCC growth by suppressing ferroptosis. Thus, rejuvenation of these tumor-suppressive ncRNAs and depression of oncogenic ncRNAs may be beneficial for HCC treatment. Moreover, it should be noted that ncRNAs-regulating ferroptosis in HCC progression is intricate due to its interaction with various pathological processes. The interplay between ncRNA-regulated ferroptosis and HCC pathogenesis should be further investigated.

### Ferroptosis-related lncRNAs as prognosis markers in HCC

LncRNAs play a crucial role in the occurrence and development of HCC by regulating ferroptosis. Screening the ferroptosis-related lncRNAs can provide an effective predictive model in HCC diagnosis, treatment, and prognosis. Xie et al. (2022) prognostic model based on the ferroptosis-related lncRNA signature may improve the survival prediction of HCC through making a classification in tumors. These ferroptosis-related lncRNAs play an essential role in the immunosuppressive tumor microenvironment, genome instability, and clinical treatment response of HCC, which may benefit to determine the individualized prognosis and treatment for HCC patients (Li et al., 2022; Lian et al., 2022). Wang D. et al. (2022) also established a ferroptosis-related lncRNA model that could accurately predict the prognosis of HCC, which is associated with tumor grade and infiltration of macrophages and fibroblasts. Further analysis revealed that these prognostic models may regulate HCC immune microenvironment via modulating immune-related pathways, such as the TNF-α/NF-κB and IL-2/STAT5 signaling pathways, which further affects the differentiation of immune infiltration (Xu Z. et al., 2021; Yang X. et al., 2022). There are other prediction models showing ferroptosis-related lncRNAs associated with immunity, tumor microenvironment alteration, chemotherapeutic, and immunotherapeutic efficacy in HCC (Fang et al., 2022; Xiong et al., 2022). Besides, 17 ferroptosis-associated lncRNAs are identified as a prognostic and risk model for accurate decision making in clinical treatment and immunotherapy of HCC (Lin and Yang, 2022). Importantly, as a key lncRNA that involved in HCC progression, MALAT1 (metastasis-associated lung adenocarcinoma transcript 1) can affect cell proliferation, apoptosis, and migration (Lu et al., 2021). It has been verified that MALAT1 is overexpressed in HCC and is associated with shorter overall survival, serving as a prognostic marker for patients with HCC (Liao et al., 2022). However, whether MALAT1 interacts with ferroptosis-related signaling pathways to regulate HCC biological function is needed to be further investigated. Therefore, these results imply that ferroptosis-related lncRNAs can be employed as promising markers for the progression, prognosis, personalized treatment, and drug resistance of HCC.

## Conclusion

**FIGURE 3:**
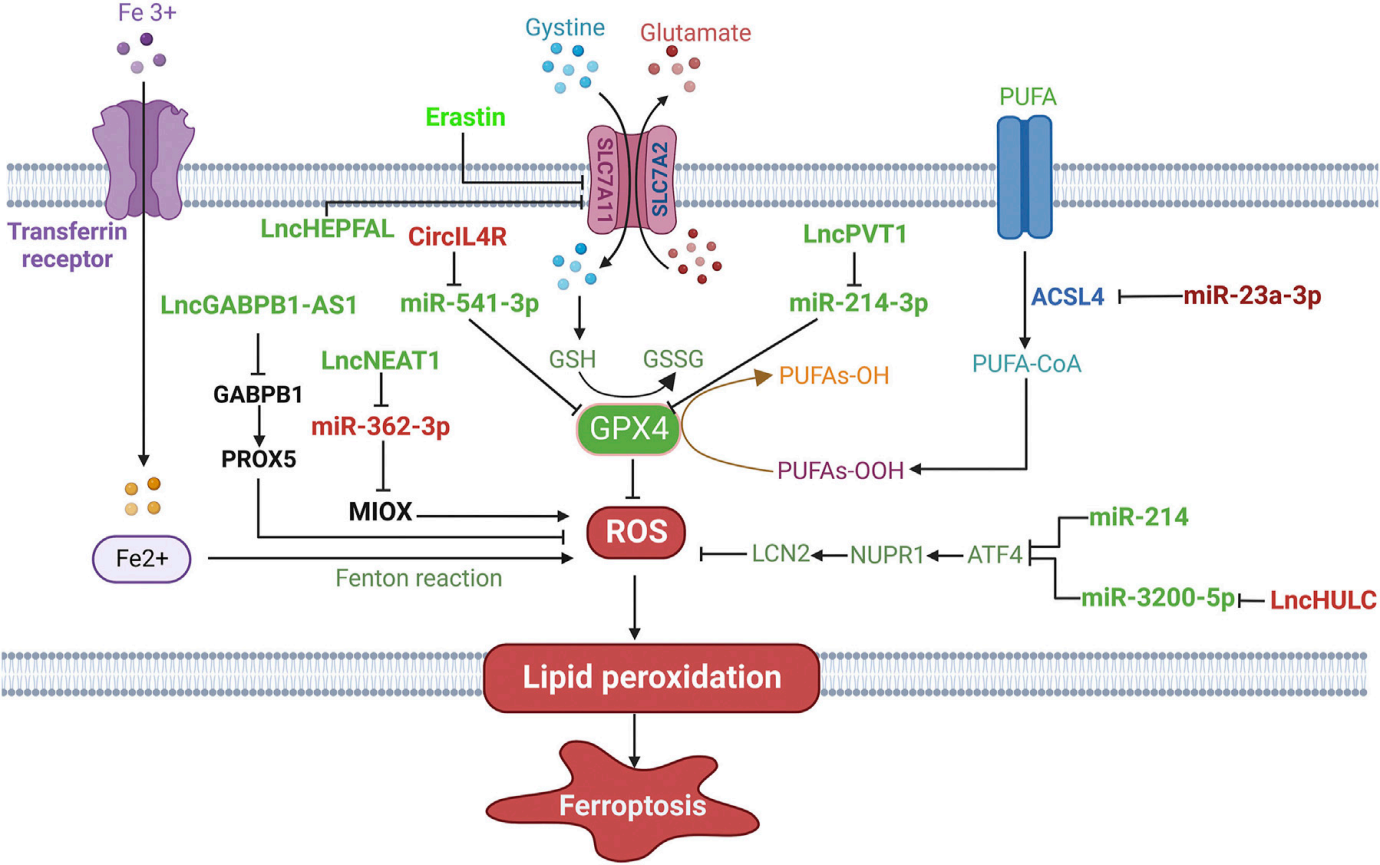
The regulatory role of ncRNAs on ferroptosis in HCC. Ferroptosis-promoting ncRNAs (green), such as lncHEPFAL, lncGABPB1-AS1 and lncNEAT1, regulate ferroptosis-related signaling pathways and components to induce ferroptosis. Ferroptosis-suppressive ncRNAs (red), including lncHULC, miR-23a-3p and cirIL4R, inhibit ferroptosis through targeting the regulatory pathways of ferroptosis. → indicates a promoting effect and ⊥ indicates an inhibitory effect.

This review summarizes the role of ferroptosis in progression, treatment, chemoresistance, and prognosis of HCC, as well as the regulatory role of ncRNAs in ferroptosis that implies a therapeutical potential for this disease. Ferroptosis induction is believed to suppress the initiation and progression of HCC, and various natural components are effective to trigger ferroptosis of HCC. As a widely used chemotherapeutic agents in HCC, sorafenib resistance poses a major hurdle. Ferroptosis activators or targeting ferroptosis-suppressors may attenuate HCC cell resistance to sorafenib. Thus, further clarifying the relationship between ferroptosis and HCC, as well as elucidating the regulatory mechanism of ferroptosis are expected to develop new tactics for molecular targeted therapies of HCC. Besides, ferroptosis-related proteins can be used as prognostic indicators for patients with HCC, but it is challengeable to identify the best candidates for the accurate prognosis. In this context, ferroptosis-related gene signature is established to evaluate the survival of patients during HCC progression and treatment. In addition, emerging ncRNAs is demonstrated to influence malignant phenotypes and chemoresistance in HCC by modulating ferroptosis (Figure 3). A comprehensive understanding of the regulatory mechanism of ncRNAs on ferroptosis in the development and treatment of HCC would help in developing effective therapeutic targets to repress and even reverse drug resistance through combined treatment. For example, in combination chemotherapeutic drugs with ferroptosis-inducing ncRNAs or downregulation of ferroptosis-suppressive ncRNAs could suppress chemoresistance and eradicate HCC. Of importance, the relationship between ferroptosis and other types of programmed cell death is still unclear. As mentioned before, ferroptosis is a novel strategy for the remedy of apoptosis resistance, so it should be paid more attention to the crosstalk between ferroptosis and other types of programmed cell death. Furthermore, utilizing the ferroptosis-associated genes or lncRNA alone to predict immune response and prognosis is insufficient. Further studies should be performed to explore how to combine ferroptosis signatures with other prognostic factors to improve the prognostic efficacy. As for the TME in HCC, further identifying the communication between immune cells and cancer cells, may provide new insights into the regulatory effects of ferroptosis on TME status, which can benefit for the design of novel drugs and combination therapies.
